# Multidrug-Resistant *Escherichia coli* Remains Susceptible to Metal Ions and Graphene-Based Compounds

**DOI:** 10.3390/antibiotics13050381

**Published:** 2024-04-24

**Authors:** Nathalie Karaky, Shiying Tang, Parameshwari Ramalingam, Andrew Kirby, Andrew J. McBain, Craig E. Banks, Kathryn A. Whitehead

**Affiliations:** 1 Great Ormond Street Institute of Child Health, University College London, London WC1N 1EH, UK; n.karaky@ucl.ac.uk; 2 Microbiology at Interfaces Group, Manchester Metropolitan University, Chester Street, Manchester M1 5GD, UK; s.tang@mmu.ac.uk; 3 Faculty of Science and Engineering, Manchester Metropolitan University, Chester Street, Manchester M1 5GD, UK; parameshwari.r@ist.srmtrichy.edu.in (P.R.); c.banks@mmu.ac.uk (C.E.B.); 4 Department of Physics, Faculty of Engineering and Technology, SRM Institute of Science and Technology, Tiruchirappalli Campus, Tiruchirappalli 620024, India; 5 Faculty of Medicine and Health, University of Leeds, Leeds LS2 9JT, UK; a.kirby@leeds.ac.uk; 6 Division of Pharmacy and Optometry, Faculty of Biology, Medicine and Health, The University of Manchester, Oxford Road, Manchester M13 9PT, UK; andrew.mcbain@manchester.ac.uk

**Keywords:** graphene, graphene oxide, metal ions, multidrug resistance, synergy, *Escherichia coli*

## Abstract

*Escherichia coli* is listed as a priority 1 pathogen on the World Health Organization (WHO) priority pathogen list. For this list of pathogens, new antibiotics are urgently needed to control the emergence and spread of multidrug-resistant strains. This study assessed eighteen metal ions, graphene, and graphene oxide for their antimicrobial efficacy against *E. coli* in both planktonic and biofilm growth states and the potential synergy between metal ions and graphene-based compounds. Molybdenum and tin ions exhibited the greatest antimicrobial activity against the planktonic states of the isolates with minimal inhibitory concentrations (MIC) ranging between 13 mg/L and 15.6 mg/L. Graphene oxide had no antimicrobial effect against any of the isolates, while graphene showed a moderate effect against *E. coli* (MIC, 62.5 mg/L). Combinations of metal ions and graphene-based compounds including tin–graphene, tin–graphene oxide, gold–graphene, platinum–graphene, and platinum–graphene oxide exhibited a synergistic antimicrobial effect (FIC ≤ 0.5), inhibiting the planktonic and biofilm formation of the isolates regardless of their antibiotic-resistant profiles. The bactericidal effect of the metal ions and the synergistic effects when combined with graphene/graphene oxide against medically relevant pathogens demonstrated that the antimicrobial efficacy was increased. Hence, such agents may potentially be used in the production of novel antimicrobial/antiseptic agents.

## 1. Introduction

Antimicrobial resistance (AMR) poses a serious threat to public health, as current antibiotics are becoming less effective in the prevention and treatment of infectious diseases worldwide [1]. Increasing reports of multidrug-resistant (MDR) bacteria depict a significant burden on the healthcare system, as MDR infections are associated with increased mortality rates, higher risk of complications and longer hospital stays resulting in huge financial strain [2]. *Escherichia coli* (*E. coli*) is one of the most clinically important MDR Gram-negative bacteria (MDR-GNB), responsible for a wide range of nosocomial infections including urinary tract infections and ventilator-associated pneumonia [3]. According to the World Health Organization (WHO) classification, *E. coli* is one of the most critical bacterial species urgently requiring new antimicrobials to address its resistance towards most of the commercially available drugs [4]. Hence, finding an alternative antimicrobial agent is vital in the race against AMR.

The use of metal ions and graphene materials with antibacterial properties is one avenue of possible exploration. The antimicrobial properties of metals have been known and used since 1500 BC in medicine and agriculture. For instance, copper was used to decontaminate drinking water and sterilise intrauterine devices [5], while silver has commonly been used in antisepsis for chronic wounds, serious burns, warts, and eye infections [6]. While it is established that toxic doses of metals selectively disrupt the processes needed for cellular growth, their use within clinical microbiology has been limited after the discovery of antibiotics. However, following the current worldwide antibiotic crisis, the possibility of using metals and metal-based components as antimicrobials and biocidal agents is again being considered [7]. The antimicrobial activity of metals and metal ions is attributed to the ability of these metal ions to interfere with DNA through covalent bonding. Metal ions are known to inhibit key enzymes by imitating the substrate. Other specific modes of metal ion action (Ag^+^, Cu^2+^, Zn^2+^) encompass various mechanisms, including the generation of oxidative stress through the production of reactive oxygen species (ROS), leading to cell damage [8]. Other metal alloys containing silver or copper ions display high antimicrobial activity by altering the pH of the physiological solution following the fast degradation of magnesium or copper alloy and the formation of Mg(OH)_2_ or Cu(OH)_2_ [8].

For the last decade, carbon nanostructures (CNS) have gained a significant attention due to their properties, allowing them to be applied in a vast number of applications including biomaterials, biosensors, and drug delivery systems [9,10]. Several allotropic forms of low-dimension carbon structures exist, which are dependent on the architectural folding shaped by carbon atoms, including fullerene, nanotubes, graphene, and diamond-like carbon [9]. Graphene is a single-atom-thick layer of graphite packed in a honeycomb two-dimensional lattice that has advantageous properties of high electrical and thermal conductivity, low light absorption, ambipolarity, and high physical strength [11]. Moreover, it has been reported that graphene nanosheets show low toxicity levels towards mammalian cells; this is an added advantage for the use of graphene in bio-related applications [12]. Several studies have shown that graphene-based materials acquire antimicrobial properties against pathogenic microorganisms [13,14]. The exact mechanism by which graphene nanomaterials interact with bacteria is still under investigation. However, several scenarios have been proposed to understand the antimicrobial effect of graphene, such as disruption of the cell membrane, removal of phospholipids from lipid membranes, and oxidative stress via ROS-dependent and independent mechanisms [15,16]. The distinctive physiochemical properties of graphene and its derivatives have sparked broad interest in biomedical research. Recent advancements in the synthesis of graphene materials have increased their accessibility in today’s market, generating heightened interest in their biomedical applications. These include serving as antimicrobial agents for tooth and bone implants, contributing to anticancer therapy, enabling the biofunctionalization of proteins, and facilitating drug delivery applications [17].

Investigating the efficacy of metal ions and graphene-based compounds as antimicrobial agents could alleviate the current burden of AMR. In this study, the antimicrobial activities of eighteen metal ions, graphene (Gr), and graphene oxide (GO) individually and in combination against MDR strains of *E. coli* with different antibiotic profiles were determined.

## 2. Results

### 2.1. Antimicrobial Susceptibility Testing

The bacterial isolates displayed different antibiotic susceptibility patterns but were both shown to be MDR. The *E. coli*-1 strain was sensitive to colistin sulphate and chloramphenicol, while being resistant to the other six antibiotics, while *E. coli*-2 strain showed resistance only to streptomycin, chloramphenicol, and tetracycline (Table 1).

### 2.2. Minimal Inhibitory Concentrations (MIC)

Eight out of the eighteen metal ions tested showed good bacteriostatic activity (MIC ≤ 31.3 mg/L) against the bacterial strains (Table 2). Molybdenum (MIC = 13 mg/L) and tin (MIC = 15 mg/L) displayed the greatest inhibitory effect against both *E. coli*. A good antimicrobial effect was also demonstrated by gold, gallium, palladium rhenium, platinum, and rhodium at 26–31.2 mg/L. However, when the antimicrobial efficacy of the acid was also taken into account, Pt, Au, Pd, and Mo all demonstrated 79.2% improvement, followed by Rh and Sn at 75.0%. The antimicrobial outcome of the rest of the tested compounds including yttrium (52.1 mg/L), ruthenium (41.6 mg/L), zirconium, niobium, tantalum, indium, zinc, and aluminium (62.5 mg/L) ions was less apparent, while a low effect was demonstrated for the silver ions (MIC = 104 mg/L) and copper ions (MIC = 125 mg/L). Graphene revealed a moderate level of antimicrobial efficacy (MIC = 62.5 mg/L) when compared to metal ions tested alone, while graphene oxide (MIC > 500 mg/L) showed no antimicrobial activity. No differences between the MICs of the metal ions and graphene were observed between the distinct isolates of the same bacterial species, despite each strain presenting a distinct antibiotic susceptibility profile.

### 2.3. Minimal Bactericidal Concentrations (MBC)

Tin, molybdenum (MBC = 26.04 mg/L), palladium (MBC = 31.2 mg/L), gallium, and gold (MBC = 41.6 mg/L), which showed a good inhibitory activity against the bacterial strains, also exhibited the greatest bactericidal activity against both isolates (Table 2). When their efficacy was taken in consideration with the effects of the acids, Pd was the best with a 75.0% improvement, followed by Ga and Au at 66.7%, and Mo and Sn at 58.3%. Platinum (52.1 mg/L), rhenium, rhodium, aluminium (MBC = 62.5 mg/L), and tantalum (MBC = 83.3 mg/L) exhibited moderate activity towards the *E. coli* strains (Table 2). The MBCs of each of niobium, zirconium, ruthenium, and silver at 125 mg/L demonstrated a lower moderate biocidal action compared to the other metal ion solutions. A lower biocidal effect was noted for indium, zinc, and copper (MBC = 250 mg/L). The MBCs of graphene oxide could not be determined (MBC > 500 mg/L) and, hence, they were classified as demonstrating no antimicrobial activity, while graphene showed a moderate antimicrobial activity at 125 mg/L. Despite the differences shown in their antibiotic profiles, both strains of each bacterium (*E. coli*-1 and *E. coli*-2) exhibited the same MBC values for each metal ion, graphene, and graphene oxide.

**Table 2:** MIC values (±SD) of metal ions, graphene, and graphene oxide against the bacterial strains. The highlighted data demonstrate the optimal results.

	MIC			MBC	
Metal Ion/Compound	Solvent	*E. coli*-1	*E. coli*-2	*E. coli*-1	*E. coli*-2
Y	HNO_3_ (2%)	52.1 ± 10.4	52.1 ± 10.4	250 ± 0.00	125 ± 0.00
Zr		62.5 ± 0.00	62.5 ± 0.00	125 ± 0.00	125 ± 0.00
Nb		62.5 ± 0.00	62.5 ± 0.00	125 ± 0.00	125 ± 0.00
Ag		104.1 ± 36.08	104.1 ± 36.08	125 ± 0.00	125 ± 0.00
Ta		62.5 ± 0.00	62.5 ± 0.00	83.3 ± 20.8	83.3 ± 20.8
In		62.5 ± 0.00	62.5 ± 0.00	250 ± 0.00	250 ± 0.00
Al		62.5 ± 0.00	62.5 ± 0.00	62.5 ± 0.00	62.5 ± 0.00
Cu		125 ± 0.00	125 ± 0.00	250 ± 0.00	250 ± 0.00
Zn		62.5 ± 0.00	62.5 ± 0.00	250 ± 0.00	250 ± 0.00
Re		31.2 ± 0.00	26.0 ± 5.20	62.5 ± 0.00	62.5 ± 0.00
Ga	HNO_3_ (5%)	26.0 ± 0.00	26.0 ± 0.00	41.6 ± 18.04	41.6 ± 18.04
Ru	HCl (5%)	41.6 ± 10.4	41.6 ± 10.4	125 ± 0.00	125 ± 0.00
Rh		31.2 ± 0.00	31.2 ± 0.00	62.5 ± 0.00	62.5 ± 0.00
Pt		26.0 ± 5.20	31.2 ± 0.00	52.1 ± 10.4	52.1 ± 10.4
Au		26.0 ± 5.20	26.0 ± 5.20	41.6 ± 18.04	41.6 ± 18.04
Pd		26.0 ± 5.20	26.0 ± 5.20	31.2 ± 0.00	31.2 ± 0.00
Mo	HCl (10%)	13.0 ± 2.60	13.0 ± 2.60	26.04 ± 5.20	26.04 ± 5.20
Sn		15.6 ± 0.00	15.6 ± 0.00	26.04 ± 5.20	26.04 ± 5.20
HNO_3_ (2%)		62.5 ± 0.00	62.5 ± 0.00	125 ± 62.5	125 ± 62.5
HNO_3_ (5%)		41.6 ± 10.4	41.6 ± 10.4	125 ± 0.00	125 ± 0.00
HCl (5%)		125 ± 0.00	125 ± 0.00	125 ± 0.00	125 ± 0.00
HCl (10%)		62.5 ± 0.00	62.5 ± 0.00	62.5 ± 0.00	62.5 ± 0.00
Graphene		62.5 ± 0.00	62.5 ± 0.00	125 ± 0.00	125 ± 0.00
Graphene oxide		>500	>500	>500	>500

### 2.4. Fractional Inhibitory Concentrations

Out of the eighteen metal ions, eight showed enhanced activity upon addition to the graphene-based compounds when used in a 1:1 ratio (Table 3 and Table 4). When combined with graphene oxide, molybdenum, tin, platinum, and ruthenium ions showed synergistic antimicrobial activity against both *E. coli* isolates, while gold, silver, yttrium, and palladium ions demonstrated an additive effect (0.5 < FIC ≤ 1). The addition of graphene exhibited a synergistic effect with molybdenum, gold, tin, and platinum ions against the two strains of *E. coli* (FIC ≤ 0.5) and an additive effect with silver, yttrium, palladium, and ruthenium ions. An additive effect was seen against both *E. coli* species when silver, yttrium, and palladium ions were combined with graphene (Table 3 and Table 4). The rest of the metal ion solutions (zirconium, niobium, tantalum, indium, aluminium, copper, zinc, rhenium, gallium, rhodium) showed an indifferent effect (1 < FIC < 4) when tested with either graphene or graphene oxide.

The most antimicrobial ions and compounds were selected for further testing. Combinations of several metal ions were tested for any variation in their antimicrobial efficacy. Sixteen (Ag-Pd, Ag-Zn, Ag-Mo, Pd-Mo, Pd-Au, Pd-Pt, Pt-Sn, Pd-Ga, Zn-Sn, Zn-Ga, Mo-Sn, Mo-Ga, Au-Pt, Au-Ga, Pd-Pt-Sn, and Au-Ag-Pd) out of the total 31 combinations tested were synergistic, showing further enhanced bacteriostatic effect (FIC ≤ 0.5) compared to metals tested alone (*p* ≤ 0.05) (Table 5). Only eight combinations (Ag-Au, Ag-Pt, Zn-Pt, Pt-Sn, Pt-Ga, Au-Sn, Au-Ga) showed an additive (0.5 < FIC ≤ 1) outcome against the *E. coli* strains, while the rest of the combinations showed an indifferent effect against both isolates (1 < FIC < 4). Both isolates showed the same affinity towards the different metal ions–graphene, metal ions–graphene oxide, or metal–metal combinations.

### 2.5. Crystal Violet Biofilm Assay

**Figure 1:**
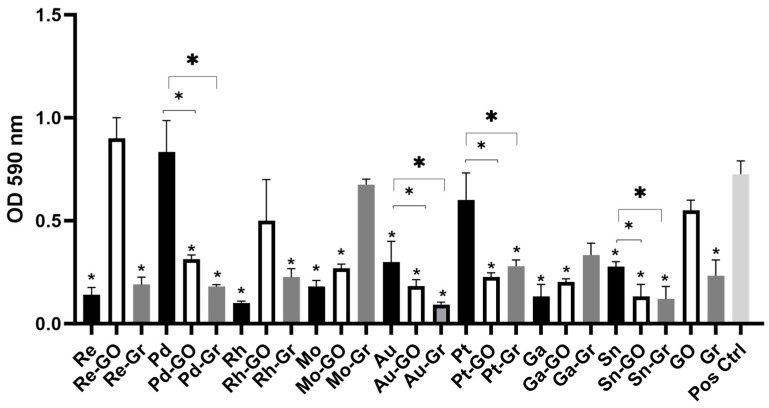
Crystal violet biofilm assay of eight metal ions tested individually or in combination with graphene or graphene oxide against representative *E. coli*. Error bars represent SD of n = 3. * signifies *p* < 0.05.

Six metal ions including rhenium, rhodium, molybdenum, gold, gallium, and tin were able to inhibit the biofilm formation of *E. coli* (Figure 1). Graphene showed an antimicrobial effect (69.4% reduction) against the biofilm forms of both bacterial strains to a greater extent than the planktonic form (*p* < 0.05) (Figure 1). The addition of Re-GO and Pd increased biofilm formation by 25.0% and 13.8%, respectively (Table 6). The metal ions alone that reduced the percentage biofilm in greater amounts than when mixed with the graphene or GO were Re (83.3%), Rh (87.5%), Mo (76.4%), and Ga (83.3%). Biofilm reduction levels for metal combinations which were also high included Re-Gr (75.0%), Pd-Gr (76.4%), Au-GO (76.4%), Au-Gr (88.9%), Sn-GO (84.7%), and Sn-Gr (86.1%). Gold ions exhibited the best antimicrobial effect when combined with graphene and were able to reduce the biofilm growth by 70% (*p* = 0.02). Other metal ions and combinations which reduced biofilm formation by >50% included Rh-Gr (70.8%), Mo-GO (61.1%), Pt-GO (70.8%), Pt-Gr (55.6%), Ga = GO (73.6%), and Ga-Gr (55.6%).

## 3. Discussion

In light of the rise of MDR bacteria leading to infections in both healthcare and community settings and contributing to higher mortality rates, it is crucial to evaluate new antibacterial agents as substitutes for traditional antimicrobial treatments. While there is a renewed interest in using metals as antimicrobial agents [7,17], this research assessed the antimicrobial properties of eighteen metal ions, graphene, and graphene oxide against MDR clinical isolates of *E. coli*.

The findings indicated that molybdenum, tin, platinum, palladium, gold, and gallium ions exhibited the greatest antimicrobial activity against the planktonic forms of *E. coli*. While a limited number of studies have explored the antimicrobial activity of metals in their ionic forms, the effect of platinum, palladium, and gold, as complexes or nanoparticles, has been demonstrated in previous studies against Gram-negative pathogens [18,19,20,21]. Ajibade and Idemudia (2013) showed the effect of Pd(II) and Pt(II) complexes of trimethoprim and pyrimethamine against *E. coli* (MIC = 10–20 mg/mL) [20]. Furthermore, Radojevic et al. synthesised complexes of Pt (IV) polymeric nanoparticles and proved that higher concentrations of 250–500 mg/mL were needed to inhibit the growth of *E. coli* ATCC 25922 [22]. On the other hand, at low concentrations of 0.1–5 µg/mL, gold nanoparticles displayed excellent antibacterial potential against *E. coli* [21].

It was evident that increasing the concentrations of the metal ions increased their bacteriostatic and bactericidal effects against the bacteria in planktonic form. The same eighteen metal ions were tested against MDR isolates of *Pseudomonas aeruginosa* in previous work and showed that platinum, palladium, and tin, followed by molybdenum, showed the highest bacteriostatic activity against the planktonic bacterial forms [23]. This might indicate that the metal ions may have different antimicrobial affinities against different pathogens. This will be further investigated in future work. The degree of antibacterial toxicity towards the bacterial cells can be influenced by the metal ion, the surrounding milieu, and the bacterium itself, in conjunction with the metal donor atom’s selectivity, reduction potential, and speciation [7].

The two bacterial isolates exhibited identical values for MIC, MBC, FIC, and CVBA, despite variations in their antibiotic profiles. This observation indicated that the metal ions effectively hindered the growth or eradicated both planktonic and biofilm cells of *E. coli*, irrespective of their antibiotic profiles. Likewise, the synergistic antibacterial impact of graphene or graphene oxide combined with metal ions remained consistent across isolates, unaffected by their antibiotic resistance patterns. These results could be attributed to the contrast in the targeted mode of action of antibiotics versus the broader mechanism of metal ions [7]. For instance, the antibacterial action of antibiotics is specific to one of four mechanisms involving inhibition or regulation of enzymes associated with cell wall biosynthesis, nucleic acid metabolism and repair, protein synthesis, or disruption of membrane structure [24]. However, the broader antibacterial scope of metal ions mainly relies on protein dysfunction, the production of reactive oxygen species, or impairment of membrane function [7]. The effectiveness of metal ions in exhibiting antibacterial properties can be initially attributed to their polarizability based on the Pearson’s theory, which classifies metals into soft and hard ions. The oxidation state of metals is influenced by various factors within the subcellular environment. Consequently, the strong reducing nature of the cytoplasm in Gram-negative bacteria, relatively compared to the periplasm, significantly affects the oxidation state of the metal and, therefore, its functionality.

It is well documented that toxic doses of these metals are capable of disrupting cell growth cycles, and this is mainly dictated by the physical and chemical properties of both the metal atoms and the accessible donor ligands within intracellular biomolecules [7]. This observation can be partially clarified by the fact that molybdenum, tin, platinum, and palladium ions, categorised as “soft metals,” exhibited the most pronounced antagonistic effects against the two isolates. The high electronegativity of these metals allows them to form covalent bonds, with a preference for the nitrogen or sulphur donors present in *E. coli* proteins. Consequently, this renders the antibacterial toxicity of these metals approximately proportional to their affinity for sulphur [25].

Surprisingly, despite its high electronegativity (1.93), silver tested in its ionic form did not show any antimicrobial activity against the planktonic form of both bacterial species in our study. This contradicted previous studies by Feng et al. (2000) that reported a mechanistic inhibition of *E. coli* by silver ions and showed significant morphological changes in the bacterial cells following treatment [26]. In a comparative study, Li et al. (2017) confirmed the antibacterial activity of silver ions using *E. coli* as a model organism. Their study also suggested that, despite having a similar mode of action, silver ions showed a better antibacterial activity than that of silver nanoparticles against bacterial cells [27]. Silver ions combined with other components such as sulfadiazine, zeolite, or nitrate have been used as broad-spectrum antimicrobials to treat infections of *Staphylococcus aureus*, *E. coli*, *P. aeruginosa*, and *Klebsiella pneumoniae*. Such silver complexes showed high antimicrobial activity against Gram-negative and Gram-positive bacteria by penetrating the cell, interfering with the replication process, binding to bacterial DNA, and leading to cell death [7]. However, concerns have arisen about bacterial resistance to silver due to its extensive and unregulated use in both medical and non-medical applications, which is thought to be set to expand to that seen for antibiotics. Silver-resistant Gram-negative pathogens (*E. coli*, *Enterobacter cloacae*, *Klebsiella pneumoniae*, and *Salmonella* spp.) have been isolated and reported. While it is still unclear if this resistance represents a threat in the clinical environment, silver resistance might pose a threat to wound and burn care in clinical settings [28,29,30].

Graphene-based compounds have emerged recently as promising materials with the potential of broad-spectrum properties [12,31]. Several studies have indicated that graphene oxide and reduced graphene oxide are able to effectively inhibit the growth of *E. coli* with at least an 86% to 99.9% reduction in viability [12,31,32,33]. This is inconsistent with our results, wherein graphene oxide did not exhibit any inhibitory or bactericidal effect against any of the isolates, since the MIC and MBC results exceeded 500 mg/L. In contrast, graphene oxide has also been demonstrated to enhance bacterial growth [34]. Therefore, the bactericidal effect of graphene-based composites remains a subject of controversy, necessitating additional analysis. This is particularly crucial as their influence on the structure and viability of bacterial cells has been demonstrated to depend on factors such as sample production and concentration, exposure time, physio-chemical properties, and the nature of the microbiological method used [12,33,35]. It could be inferred from this study that the lack of antimicrobial efficacy observed with graphene oxide might be attributed to the specific type of graphene oxide employed. This could also be attributed to the distinctive physiochemical properties of the graphene oxide, including the alignment, exposition of the functional group exposed, dispersibility, and size of the GO sheets [36]. Although graphene oxide alone exhibited no antimicrobial activity against any of the isolates, the combination of graphene oxide or graphene with molybdenum, tin, and platinum ions amplified their bacteriostatic effect.

Our results indicated that although the bactericidal effect was evident from the metal ions when used singularly against the strains, the addition of graphene or graphene oxide caused a synergistic effect. This may indicate that combinations of metal ions and graphene resulted in complementary modes of action, boosting the overall antimicrobial effect. For instance, it is known that metals inhibit bacterial growth through different chemical and physical mechanisms including protein dysfunction, membrane impairment, reactive oxygen species production, and nutrient assimilation [19]. However, a conceivable hypothesis could be that since the metal ions are in solution, they become uniformly distributed in the environment surrounding the pathogen with no specific laterality [19,37]; this will also be influenced by the chemical charges of the surrounding milieu. Meanwhile, by adsorbing to the pathogen, graphene particles are able to make the bacterial cell more permeable to destruction due to cell wall depolarisation. This enables metal ions to penetrate the bacterial cell interior [19,38]. The different modes of action of the graphene/graphene oxide particles and metal ions in combination may provide an explanation as to their synergistic effect.

While estimates suggest that approximately 65% of bacterial infections are linked to biofilms, encompassing both device-related and non-device-related infections [36], combinations of gold–graphene, platinum–graphene oxide, platinum–graphene, tin–graphene, and tin–graphene oxide inhibited both the planktonic cells and the biofilm forms of the tested strains. The potential use of such combinations might play a role in the reduction of biofilm formation, especially for GNB on surfaces being implemented in indwelling medical devices or antimicrobial cleansers that are used in clinical settings, which would lead to a decline in hospital-acquired infections [39,40].

## 4. Experimental

### 4.1. Bacterial Strains

Two clinical isolates of *E. coli* were collected from Leeds Infirmary Hospital and were evaluated in this study. Bacterial isolates were cultured on tryptone soy agar (TSA) (Oxoid, Basingstoke, UK) or tryptone soy broth (TSB) (Oxoid, Basingstoke, UK) and incubated for 24 h at 37 °C in an aerobic atmosphere. All assays were repeated in triplicate. The isolates were designated as *E. coli*-1 and *E. coli*-2.

### 4.2. Antibiotic Susceptibility Testing

All isolates were tested for antibiotic susceptibility using the disc diffusion method in accordance with standards recommended by the European Committee on Antimicrobial Susceptibility Testing (EUCAST) [16]. The *E. coli* strains were tested using multidisc (MAST, Merseyside, UK) containing the following antibiotics: ampicillin (25 µg), chloramphenicol (50 µg), colistin sulphate (100 µg), kanamycin (30 µg), nalidixic acid (30 µg), nitrofurantoin (50 µg), streptomycin (25 µg), and tetracycline (100 µg) (AB Biodisk, Cambridge, UK). Following an overnight incubation at 37 °C, the diameters of inhibition zones were measured in millimetres and interpreted in accordance with the EUCAST guidelines. Bacterial isolates were classified as MDR if they showed resistance to multiple (three or more) antimicrobial agents, classes, or subclasses of antibiotics [41]. *E. coli* NCTC 9001 was used as a control strain.

### 4.3. Antimicrobial Compounds

The metal ions examined in this study were suspended in acid solutions of hydrochloric acid (HCl) or nitric acid (HNO_3_) and included silver (Ag), aluminium (Al), copper (Cu), indium (I), niobium (Nb), rhenium (Re), tantalum (Ta), yttrium (Y), zinc (Zn), zirconium (Zr) (2% HNO_3_), gallium (Ga) (5% HNO_3_), gold (Au), palladium (Pd), platinum (Pt), ruthenium (Ru), rhodium (Rh) (5% HCl), tin (Sn), and molybdenum (Mo) (10% HCl). All solutions were at 1000 mg/L of Atomic Absorption Standards (AAS) and were purchased from Sigma-Aldrich^®^, Gillingham, Dorset, UK. Graphene oxide (GO) with flake sizes ranging from 300 nm to 700 nm in a solution of 500 mg/L (Graphene-Supermarket, Ronkonkoma, NY, USA), and graphene particles (200 nm–1 µm), synthesised at the Manchester Metropolitan University and suspended in water, were also investigated.

### 4.4. Minimum Inhibitory Concentration (MIC) and Minimum Bactericidal Concentration (MBC) Assays

Following an overnight incubation in Tryptone Soya Broth (TSB), bacterial cultures were centrifuged (1721× *g*) for 10 min. The supernatant was discarded, and the pellet was re-suspended in 10 mL of double-strength TSB containing 0.15% triphenyl blue chloride (TBC) (Sigma-Aldrich^®^, Gillingham, Dorset, UK). A hundred microliters of bacterial suspension were adjusted to an OD_600_ of 1.0 (±0.1) in sterile distilled water at 540 nm and added to an equal volume of the test compound. The last column served as a negative control and included 100 µL of double-strength TSB broth with 0.15% TBC and 100 µL of sterile water. Microplates were sealed with Parafilm^®^ (VWR, Lutterworth, Leicestershire, UK) and incubated at 37 °C for 24 h. The Minimum Inhibitory Concentration (MIC), indicating the lowest concentration of the tested metal ion, was identified as the first well showing no blue pigmentation, signifying the absence of viable cells. Minimum Bactericidal Concentrations (MBCs) were determined by plating 20 µL of each well without visible blue pigmentation onto TSA and incubating them at 37 °C for 24 h. The lowest concentration showing no bacterial growth was referred to as MBC. The correlation between MIC and MBC values, along with the scope of antimicrobial activity, was subdivided as follows for this study: good antimicrobial activity (MIC ≤ 31.25 mg/L and MBC ≤ 41.6 mg/L), moderate antimicrobial activity (31.25 mg/L < MIC ≤ 84 mg/L and 52 ≤ MBC ≤ 125), low antimicrobial activity (100 < MIC ≤ 250 and 250 ≤ MBC ≤ 500), and no antimicrobial activity (MIC or MBC > 500 mg/L).

### 4.5. Fractional Inhibitory Concentration Assay

The synergistic effects of the metal ions and graphene or graphene oxide were tested using fractional inhibitory concentration (FIC) assays following the same procedure as the MIC. The metal ions and graphene/graphene oxide were added in a 1:1 ratio. FIC ratios were calculated as previously described [17]. FIC ratios of two compounds X and Y were calculated and interpreted as follows [17,42]:

∑FIC = FIC (X) + FIC (Y)

$$\mathrm{FIC}\;\mathrm{of}\;\mathrm{compound}\;X=\frac{\mathrm{MIC}\;\mathrm{of}\;\mathrm{compound}\;X\;\mathrm{in}\;\mathrm{combination}}{\mathrm{MIC}\;\mathrm{of}\;\mathrm{compound}\;X\;\mathrm{alone}}$$

$$\mathrm{FIC}\;\mathrm{of}\;\mathrm{compound}\;Y=\frac{\mathrm{MIC}\;\mathrm{of}\;\mathrm{compound}\;Y\;\mathrm{in}\;\mathrm{combination}}{\mathrm{MIC}\;\mathrm{of}\;\mathrm{compound}\;Y\;\mathrm{alone}}$$

X and Y were considered to be synergistic if ∑FIC ≤ 0.5, additive if 0.5 < ∑FIC ≤ 1, indifferent if ∑FIC > 1, and antagonistic if ∑FIC ≥ 4.

### 4.6. Crystal Violet Biofilm Assay

Finely polished 304-grade stainless steel coupons measuring 10 mm × 10 mm were used for biofilm formation. The coupons underwent cleaning with undiluted acetone, methanol, and ethanol (BDH, Brighouse, UK) for 10 min each, with intermediate washes using sterile water. Subsequently, they were positioned at the centres of the wells in twelve-well culture plates, each containing 1 mL of a washed bacterial cell suspension suspended in TSB (OD 1.0). The plates were sealed with parafilm and then incubated for 7 days at 37 °C [23]. Following incubation, the coupons were gently washed with 2 mL of sterile distilled water to eliminate any loosely attached planktonic cells and air-dried at room temperature for 2 h. Each tested compound––metal ions, graphene, or graphene oxide (1 mL) (500 mg/L)––was introduced into the respective well containing the coupons and incubated for 24 h at 37 °C. In synergy testing involving two metals, 500 µL of each compound was used. Wells inoculated with TSB only served as negative controls. The metal ions were eliminated after incubation, and the coupons were washed with 1 mL of sterile distilled water. Crystal violet (0.03%) (Oxoid, Basingstoke, UK) was used to stain adherent cells for 30 min, then washed with sterile water and air-dried for 1 h. Subsequently, 1 mL of 33% glacial acetic acid (BDH, Brighouse, UK) was added to each well and allowed to stand for 30 min. The absorbance of the supernatant was then measured at OD_590_.

### 4.7. Statistical Analysis

Statistical analysis in this study was performed using IBM SPSS Statistics Software (version 25). The distribution of the data from mean values was analysed using standard deviation. Independent sample *t*-tests with a two-tailed distribution and one-way analysis of variance tests were performed. Data were considered significant when *p* < 0.05. 

## 5. Conclusions

This study demonstrated that gold, tin, and combinations of gold–graphene, platinum–graphene oxide, platinum–graphene, tin–graphene and tin–graphene oxide ions exhibited antimicrobial properties against the planktonic and biofilm forms of *E. coli*, irrespective of the resistance pattern of each strain. The antimicrobial effect was more enhanced when combined with graphene-based compounds. In light of the growing increase in resistance to the currently available antibiotics and to different forms of silver agents which have been consistently used as potent antibacterial agents for decades, this study suggests that metal ions and graphene derivatives present potential antimicrobial alternatives for the eradication of bacterial infections, including MDR strains. While the toxicity of metal ions remains a persistent issue, further investigations are needed to analyse the behaviour of metal ions, which may refine the selectivity towards different bacterial cells.

## Figures and Tables

**Table 1:** Zones of inhibition (mm) and antibiotic profiles for the two *E. coli* strains used in this study.

Antibiotic	*E. coli*-1	*E. coli*-2
Ampicillin	19.33 ± 1.52	11.00 ± 1.00
Chloramphenicol	12.33 ± 0.57	22.00 ± 1.73
Colistin sulphate	23.66 ± 1.15	27.33 ± 1.52
Kanamycin	13.00 ± 1.00	26.66 ± 1.52
Nalidixic acid	15.00 ± 1.00	24.66 ± 0.57
Nitrofurantoin	6.00 ± 1.00	17.66 ± 1.15
Streptomycin	10.33 ± 1.00	11.33 ± 1.52
Tetracycline	9.66 ± 0.57	7.33 ± 0.57

**Table 3:** FIC indices of combinations of metal ions and graphene oxide against bacterial isolates of *E. coli*. The highlighted data demonstrate the optimal results.

FICs of GO (1:1)	Mo	Au	Ag	Sn	Y	Pt	Pd	Ru
*E. coli*-1	0.50	1.00	1.00	0.50	1.00	0.50	1.00	0.25
*E. coli*-2	0.45	1.00	1.00	0.47	1.00	0.49	0.99	0.19

**Table 4:** FIC indices of combinations of metal ions and graphene against bacterial isolates of *E. coli*. The highlighted data demonstrate the optimal results.

FICs of Gr (1:1)	Mo	Au	Ag	Sn	Y	Pt	Pd	Ru
*E. coli*-1	0.5	0.49	1.00	0.49	1.00	0.49	1.00	1.00
*E. coli*-2	0.33	0.33	0.75	0.50	0.99	0.50	1.00	0.85

**Table 5:** FIC values of different metal combinations against *E. coli* bacterial isolates. The light grey boxes demonstrate synergistic interactions, the dark grey boxes additive interactions and the uncoloured boxes indicate indifferent interactions.

	*E. coli*-1	*E. coli*-2
Ag + Pd	0.21	0.25
Ag + Zn	0.25	0.25
Ag + Mo	0.19	0.25
Ag + Au	1.00	1.00
Ag + Pt	1.00	1.00
Ag + Sn	3.00	2.00
Ag + Ga	1.00	1.00
Pd + Zn	3.00	2.00
Pd + Mo	0.38	0.49
Pd + Au	0.21	0.25
Pd + Pt	0.19	0.12
Pd+ Sn	0.27	0.24
Pd + Ga	0.44	0.50
Zn + Mo	3.00	2.00
Zn + Au	3.00	2.00
Zn + Pt	1.00	1.00
Zn + Sn	0.45	0.50
Zn + Ga	0.33	0.50
Mo + Au	2.00	2.00
Mo + Pt	1.00	1.00
Mo + Sn	0.33	0.49
Mo + Ga	0.50	0.50
Pt + Sn	1.00	1.00
Pt + Ga	1.00	1.00
Sn + Ga	3.00	2.00
Au + Pt	0.35	0.49
Au + Sn	1.00	0.99
Au + Ga	0.33	0.5
Au + Ag + Sn	1.00	0.99
Pd + Pt + Sn	0.19	0.25
Au + Ag + Pd	0.25	0.12

**Table 6:** Percentage biofilm reduction following the crystal violet biofilm assays tested individually or in combination with graphene or graphene oxide against representative *E. coli*.

	% Biofilm Reduction		% Biofilm Reduction		% Biofilm Reduction		% Biofilm Reduction		% Biofilm Reduction
Re	83.3	Rh	87.5	Au	59.7	Ga	83.3	GO	26.4
Re-GO	−25	Rh-GO	31.9	Au-GO	76.4	Ga-GO	73.6	Gr	69.4
Re-Gr	75	Rh-Gr	70.8	Au-Gr	88.9	Ga-Gr	55.6	Ctrl	0
Pd	−13.8	Mo	76.4	Pt	16.7	Sn	62.5		
Pd-GO	58.3	Mo-GO	61.1	Pt-GO	70.8	Sn-GO	84.7		
Pd-Gr	76.4	Mo-Gr	5.6	Pt-Gr	55.6	Sn-Gr	86.1		

## Data Availability

Data will be made available upon reasonable request to the authors.

## References

[1] Dadgostar P. (2019). Antimicrobial resistance: Implications and costs. Infect. Drug Resist..

[2] Llor C., Bjerrum L. (2014). Antimicrobial resistance: Risk associated with antibiotic overuse and initiatives to reduce the problem. Ther. Adv. Drug Saf..

[3] Ruppe E., Woerther P.-L., Barbier F. (2015). Mechanisms of antimicrobial resistance in *Gram-negative bacilli*. Ann. Intensive Care.

[4] World Health Organization (2017). Prioritization of Pathogens to Guide Discovery, Research and Development of New Antibiotics for Drug-Resistant Bacterial Infections, Including Tuberculosis.

[5] O’Gorman J., Humphreys H. (2012). Application of copper to prevent and control infection. Where are we now?. J. Hosp. Infect..

[6] Silver S., Phung T., Silver G. (2006). Silver as biocides in burn and wound dressings and bacterial resistance to silver compounds. J. Ind. Microbiol. Biotechnol..

[7] Lemire J.A., Harrison J.J., Turner R.T. (2013). Antimicrobial activity of metals: Mechanisms, molecular targets and applications. Nat. Rev. Microbiol..

[8] Waters J.E., Stevens-Cullinane L., Siebenmann L., Hess J. (2023). Recent advances in the development of metal complexes as antibacterial agents with metal-specific modes of action. Curr. Opin. Microbiol..

[9] Al-Jumaili A., Alancherry S., Bazaka K., Jacob M. (2017). Review on the antimicrobial properties of carbon nanostructures. Materials.

[10] Bitounis D., Ali-Boucettta H., Hong B.H., Min D.H., Kosteralos K. (2013). Prospects and challenges of graphene in biomedical applications. Adv. Mater..

[11] Allen M.J., Tung V.C., Kaner R.B. (2010). Honeycomb carbon: A review of graphene. Chem. Rev..

[12] Hu W., Peng C., Luo W., Lv M., Li X., Li D., Huang Q., Fan C. (2010). Graphene-based antibacterial paper. ACS Nano.

[13] Dizaj S.M., Mennati A., Jarafi S., Khezri K., Adibkia K. (2015). Antimicrobial activity of carbon-based nanoparticles. Adv. Pharm. Bull..

[14] Ji H., Sun H., Qu X. (2016). Antibacterial applications of graphene-based nanomaterials: Recent achievements and challenges. Adv. Drug Deliv. Rev..

[15] Sanchez V.C., Jachak A., Hurt R.H., Kane A.B. (2012). Biological interactions of graphene-family nanomaterials: An interdisciplinary review. Chem. Res. Toxicol..

[16] Zhou R., Gao H. (2014). Cytotoxicity of graphene: Recent advances and future perspective. Wiley Interdiscip. Rev. Nanomed. Nanobiotechnol..

[17] Mohammed H., Ajay Kumar A., Bekyarova E., Al-Hadeethi Y., Zhang Y., Chen m., Ansari M.S., Cochis A., Rimondini L. (2020). Antimicrobial mechanisms and effectiveness of graphene and graphene-functionalized biomaterials. A scope review. Front. Bioeng. Biotechnol..

[18] Lai H.Z., Chen W.Y., Wu C.Y., Chen Y.C. (2015). Potent antibacterial nanoparticles for *Pathogenic bacteria*. ACS Appl. Mater. Interfaces.

[19] Slavin Y.N., Asnis J., Häfeli U.O., Bach H. (2017). Metal nanoparticles: Understanding the mechanisms behind antibacterial activity. J. Biotechnol..

[20] Ajibade P.A., Idemudia O.G. (2013). Synthesis, characterization and antibacterial studies of Pd (II) and Pt (II) complexes of some diaminopyrimidine derivatives. Bioinorg. Chem. Appl..

[21] Zhou Y., Kong Y., Kundu S., Cirillo J.D., Liang H. (2012). Antibacterial activities of gold and silver nanoparticles against *Escherichia coli* and bacillus Calmette-Guerin. J. Nanobiotechnol..

[22] Radojevic I., Sava V., Ljiljana Č., Srećko T., Marina M., Miloš N., Gordana R. (2017). Antibacterial and antibiofilm screening of new platinum (IV) complexes with some s-alkyl derivatives of *Thiosalicylic acid*. Kragujev. J. Sci..

[23] Karaky N., Kirby A., McBain A.J., Butler J.A., El Mohtadi M., Banks C.E., Whitehead K.A. (2020). Metal ions and graphene-based compounds as alternative options for burn wounds infected by antibiotic-resistant *Pseudomonas aeruginosa*. Arch. Microbiol..

[24] Kohanski M.A., Dwyer D.J., Collins J.J. (2010). How antibiotics kill bacteria: From targets to networks. Nat. Rev. Microbiol..

[25] Harrison J.J., Ceri H., Turner R.J. (2007). Multimetal resistance and tolerance in microbial biofilms. Nat. Rev. Microbiol..

[26] Feng Q.L., Wu J., Chen G.Q., Cui F.Z., Kim T.N., Kim J.O. (2000). A mechanistic study of the antibacterial effect of silver ions on *Escherichia coli* and *Staphylococcus aureus*. J. Biomed. Mater..

[27] Li W.R., Sun T.L., Zhou S.L., Ma Y.K., Shi Q.S., Xie X.B. (2017). A comparative analysis of antibacterial activity, dynamics, and effects of silver ions and silver nanoparticles against four bacterial strains. Int. Biodeterior. Biodegrad..

[28] Atiyeh B.S., Costagliola M., Hayek S.N., Dibo S.A. (2007). Effect of silver on burn wound infection control and healing: Review of the literature. Burns.

[29] Percival S.L., Bowler P.G., Russel D. (2005). Bacterial resistance to silver in wound care. J. Hosp. Infect..

[30] Chopra I. (2007). The increasing use of silver-based products as antimicrobial agents: A useful development or a cause for concern?. J. Antimicrob. Chemother..

[31] Tu Y., Lv M., Xiu P., Huynh T., Zhang M., Castelli M., Liu Z., Huang Q., Fan C., Fang H. (2013). Destructive extraction of phospholipids from *Escherichia coli* membranes by graphene nanosheets. Nat. Nanotechnol..

[32] Ocsoy I., Paret M.L., Ocsoy M.A., Kunwar S., Chen T., You M., Tan W. (2013). Nanotechnology in plant disease management: DNA-directed silver nanoparticles on graphene oxide as an antibacterial against *Xanthomonas perforans*. ACS Nano.

[33] Liu S., Zeng T.H., Hofmann M., Burcombe E., Wei J., Jiang R., Kong J., Chen Y. (2011). Antibacterial activity of graphite, graphite oxide, graphene oxide, and reduced graphene oxide: Membrane and oxidative stress. ACS Nano.

[34] Ruiz O.N., Fernando K.A., Wang B., Brown N.A., Luo P.G., McNamara N.D., Vangsness M., Sun Y.P., Bunker C.E. (2011). Graphene oxide: A nonspecific enhancer of cellular growth. ACS Nano.

[35] Akhavan O., Ghaderi E. (2010). Toxicity of graphene and graphene oxide nanowalls against bacteria. ACS Nano.

[36] Ng I.M., Shamsi S. (2022). Graphene Oxide (GO): A Promising Nanomaterial against Infectious Diseases Caused by Multidrug-Resistant Bacteria. Int. J. Mol. Sci..

[37] McQuillan J.S., Infante H.G., Stokes E., Shaw A.M. (2012). Silver nanoparticle enhanced silver ion stress response in *Escherichia coli* K12. Nanotoxicology.

[38] Pal S., Tak Y.K., Song J.M. (2007). Does the antibacterial activity of silver nanoparticles depend on the shape of the nanoparticle? A study of the *Gram-negative bacterium Escherichia coli*. Appl. Environ. Microbiol..

[39] Jamal M., Ahmad W., Andleeb S., Jalil F., Imran M., Nawaz M.A., Hussain T., Ali M., Rafiq M., Kamil M.A. (2018). Bacterial biofilm and associated infections. JCMA.

[40] Branda S., Vik A., Friedman L., Kiolter R. (2005). Biofilms: The matrix revised. Trends Microbiol..

[41] European Committee on Antimicrobial Susceptibility Testing (EUCAST) (2008). Breakpoint Tables for Interpretation of MICs and Zone Diameters. https://www.eucast.org/clinical_breakpoints.

[42] Magiorakos A.P., Srinivasan A., Carey R.B., Carmeli Y., Falagas M.E., Giske C.G., Harbarth S., Hindler J.F., Kahlmeter G., Olsson-Liljequist B. (2012). Multidrug-resistant, extensively drug-resistant and pandrug-resistant bacteria: An international expert proposal for interim standard definitions for acquired resistance. Clin. Microbiol. Infect..

